# Editorial: Entrepreneurship and Digital Transformation: Managing Disruptive Innovation in a Changing Environment

**DOI:** 10.3389/fpsyg.2021.735503

**Published:** 2021-08-13

**Authors:** Victor García-Morales, Aurora Garrido-Moreno, Rodrigo Martín-Rojas, Nigel Lockett

**Affiliations:** ^1^Faculty of Economics and Business, University of Granada, Granada, Spain; ^2^Faculty of Social Studies and Social Work, University of Malaga, Málaga, Spain; ^3^Hunter Centre for Entrepreneurship, University of Strathclyde, Glasgow, United Kingdom

**Keywords:** entrepreneurship, digital transformation, social media, COVID-19, disruptive innovation

The advent of digital technologies and the current environmental turbulence is fundamentally changing the way firms compete, eroding firm's boundaries and transforming value creation processes (Jonsson et al., 2018). The digital transformation invokes visions, ranging from the disruption of entire industries to the rethinking of its fundamental business model or its place in the value chain. This digital transformation encompasses the process of using digital technologies to create new business processes, new entrepreneurial activities, or modify the existing ones, culture and customer experiences to meet changing business and market requirements (Ransbotham et al., 2016).

There is no doubt that digital technologies have the potential to foster disruptive innovation and disruptive entrepreneurial activity in a wide range of sectors, both in manufacturing and services, as well as in commercial, educational and social domains (Christensen et al., 2006). In spite of the current relevance of disruptive innovation in academic and business circles, a better understanding of the phenomenon, which help firms to successfully innovate, is needed.

In this regard, the current Research Topic encompass a body of work including contributions from a total of 50 authors of different universities from 11 countries. Therefore, we can affirm it has a global character, collecting research works from three different continents: Europe (Germany, Italy, Lithuania, Romania, Slovenia, Spain, UK); Asia (China, Pakistan, Singapore) and Oceania (Australia). This collection is composed by 14 original research papers, examining three main topics: (1) *analysis of entrepreneurship and entrepreneurial education*; (2) *digital transformation and digital technologies* (including *Social Media tools*); and (3) *disruptive innovation and disruptive impact of Covid-19*. In next paragraphs, we describe briefly the different studies, grouped in the aforementioned thematic blocks.

First, different authors explored the topic of *entrepreneurship and entrepreneurial education*. In this vein, Junaid et al. examined the impact of informal institutions in promoting entrepreneurial activities. Examining data from 56 countries, results confirm how institutional antecedents combine distinctly for men's and women's entrepreneurship and this combination varies in countries with different stages of economic development. Kumpikaité-Valiuniené et al. focused its analysis in the figure of “expat-preneurs,” examining the main factors that lead expatriates to develop entrepreneurial activities in foreign countries. Findings display the main demographic characteristics and motivation of expat-preneurs in a Lithuanian context. Additionally, several papers focused in the phenomenon of entrepreneurial education, provident relevant guidance and practical implications for institutions. For example, Ma et al. drawing on Fuzzy-DEMATEL and ISM methods developed a hierarchical framework for the application of big data technology in entrepreneurship education, which can be helpful for managers to organize educational activities from a macro perspective. Pérez-Fernández et al. empirically examined the role that social and psychological factors play in fostering entrepreneurial activities. Focusing on a sample of higher education students in Spain, results confirm the impact of online and face-to-face social networks, as well as positive dispositional affectivity on students' entrepreneurial intention. Finally, Martínez-Martínez and Ventura quantitatively examined the key role of entrepreneurial competences among students, providing a useful classification of entrepreneurial profiles. Findings highlight the relevance of networking and professional social skills, community engagement, or perseverance of effort, and offer interesting implications for universities, to foster entrepreneurial education.

Second, considering the current relevance of *digital transformation and digital technologies*, several papers of the Research Topic examined the issue. From a theoretical perspective, Vaska et al. conducted a structured review of the literature analyzing the development of the digital transformation field, and exploring the impact of digital technologies on business model innovation. Results describe the state of this emerging research field and provide interesting avenues for future research. From a more practical perspective, to explore the process of firms' adoption of digital strategies, Aramburu et al. empirically examined how SMEs develop digitalization processes, and the main capabilities involved. Findings confirm that digital mindset, digital mindset and empowered employees are key factors to develop a digitally enabled growth strategy. In the same vein, Roblek et al. explored the impact of digital transformation on manufacturing SMEs, and highlight key success factors to conduct disruptive innovations, offering relevant implications for practitioners.

Other articles particularly focused on the *analysis of Social Media tools*, as they are considered key techonologies enabling business transformation and digitalization (Aral et al., 2013), which have disrupted entire industries. In this vein, Popescul et al. theoretically examined the success factors of Social Media-based crowdfunding campaigns for start-up projects, and highlight the main determinants of investors decisions, providing practical implications for platforms' managers. Drawing on the current relevance of Social Media use and electronic word-of-mouth (eWOM) for firms, Sánchez-González and González-Fernández empirically examined the antecedents of eWOM in the hotel industry. Findings empirically display the variables that promote customer participation in Social Media communication processes and offer strategic recommendations for hotel managers. Additionally, Rodríguez-Gómez et al. explored the phenomenon of Social Media use as relevant learning tool, and empirically observed how the use of methods based on Web 2.0 and Social media tools are useful to teach ethics and Corporate Social Responsibility to undergraduate students.

Finally, focusing on disruptive innovations, we should highlight that, during the process of production of the current Research Topic (2020), an unexpected phenomenon, as was the irruption of Covid-19 pandemic, caused a global disruption, that affected and transformed all aspects of our daily life. Some papers of the issue addressed the phenomenon, examining the disruptive impact of Covid-19 in aspects such as university education or in the transformation of workplaces, with the emergence of teleworking. For example, García-Morales et al. examined the digital transformation of higher education after Covid-19 disruption, describing main barriers and challenges that universities found during this transformative process and highlighting also main technologies and methodologies used to evolve to online teaching in an extremely short time. Likar and Trcek applied a novel method of innovation management techniques to examine evolving challenges that arise in the transformation of higher education processes, offering interesting implications to improve distance learning processes. Considering that the arrival of Covid-19 dramatically accelerates firms' digitalization trends, Trenerry et al. identified the main categories of factors that are essential to enable an effective digital transformation of workplaces in the current scenario.

In sum, based in all the above we can affirm that this Research Topic conforms an eclectic and integrative work, empirically addressing different sectors, from education to hospitality, and examining key concepts like business model innovation, Human Resources Management, entrepreneurial education, and Social Media use. The papers included in this collection clearly contribute to extend literature in fields such as entrepreneurship; digitalization, digital transformation, and technologies and disruptive innovation. Given the fact that pandemic situation impacted on the middle of this special issue, we also included in the Research diverse studies related to the irruption of Covid-19, and its disruptive effect in different areas.

In conclusion, the current Research Topic includes 14 research papers examined the mentioned topics form different perspectives, and providing interesting theoretical and practical insights to reserarchers and practitioners working in the fields of entrepreneurship and digital transformation. Taking into account the diversity and breadth of the topics analyzed, we are aware that the current work does not embrace all the different perspective and facets of the topics. Therefore, we suggest that more research efforts should be directed to digital environment and the dynamic and connective tools in strategy management (Ransbotham et al., 2016), so that organizations may gain valuable knowledge to drive innovation processes and firm performance. Moreover, different research approaches, such as meta-analysis, multi-case study, comparative international entrepreneurship, and mix-method research, are also recommended so as to cast light on entrepreneurship and its impacts on education and society.

## Author Contributions

All authors equally contributed to the manuscript and approved the submitted version.

## Conflict of Interest

The authors declare that the research was conducted in the absence of any commercial or financial relationships that could be construed as a potential conflict of interest.

## Publisher's Note

All claims expressed in this article are solely those of the authors and do not necessarily represent those of their affiliated organizations, or those of the publisher, the editors and the reviewers. Any product that may be evaluated in this article, or claim that may be made by its manufacturer, is not guaranteed or endorsed by the publisher.
